# Obstetric Fistula in Ilorin, Nigeria

**DOI:** 10.1371/journal.pmed.0010002

**Published:** 2004-10-19

**Authors:** Andrew Browning

## Abstract

In this perspective, Andrew Browning of the Fistula Hospital in Addis Ababa discusses a study on obstetric fistula in Ilorin, Nigeria. The study was originally published in the *West African Journal of Medicine* [1]. With the journal's permission, we have made a PDF of the full-text article freely available on our website (see Text S1).

## Abstract 2

Obstetric fistula causes women enormous distress and social isolation in developing countries like Nigeria. What do we know about the condition?

The obstetric urogenital fistula has caused women misery ever since they first started delivering children. It was once common worldwide, but with the advent of safe obstetric care during the early part of the last century, the condition has become rare in rich countries. Urogenital fistulae do still occur in developed countries, but unlike in the developing world, they are usually a complication of a difficult pelvic surgery, cancer, or radiation [2].

## Obstetric Fistula: A Disease of Poverty

Obstetric fistula—a urogenital fistula from obstructed labour—is now only encountered in countries where health resources are scarce. The shame associated with incontinence drives affected women further into a life of poverty and begging. Many women with fistula either do not know that they can get medical help, or if they do, they are unable to pay.

Furthermore, very little scientific research has been published about obstetric fistula and its management, partly because the people treating patients with this condition are working in remote areas, often with very limited resources for research. What has been written consists largely of personal case series and a few epidemiological studies [2].

To date there has been only one randomised trial in the developing world, involving 79 women operated on by a single surgeon in Benin, which found that intra-operative intravenous antibiotics did not reduce the risk of failed surgical repair or of objective incontinence [3]. There has been only one study in a developing country comparing different surgical techniques—a retrospective study of 46 patients operated on over a five-year period at a hospital in Mumbai, India [4]. This study suggested that a technique called the Martius procedure (which involves grafting of a labial pad of fat) may be better than simple anatomic repair.

What we do know about the obstetric urogenital fistula is that the women who have these injuries are young, usually illiterate, and of a lower socioeconomic background. They are more often primiparous and short in stature, and they have an average length of labour of some 3.9 days. The labour is usually unattended, or if attended, it is by someone unskilled. The women inevitably deliver a stillborn child. About half of the women with fistula are divorced as a direct result of their incontinence [2,5,6,7].

## The Injury and its Consequences

The initial injury that leads to a fistula results from ischaemic necrosis of the soft tissues of the pelvis due to an impacted presenting part during the long labour.[Fig pmed-0010002-g001]

**Figure pmed-0010002-g001:**
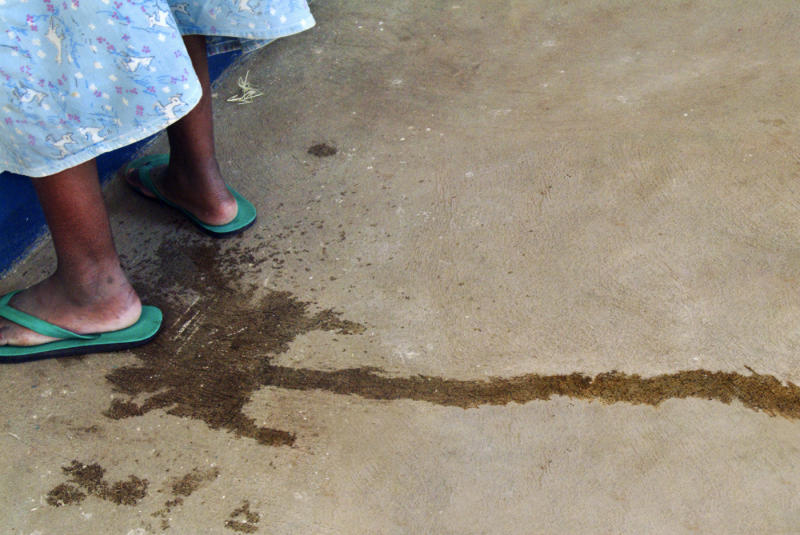
Incontinent women face a life of shame and isolation (Photograph: © 2004 Shaleece Haas. This is an open-access image distributed under the terms of the Creative Commons Attribution License, which permits unrestricted use, distribution, and reproduction in any medium, provided the original work is properly cited.)

The ischaemia then affects the bladder and vagina (and sometimes the rectum and vagina), resulting in a fistula. The process also affects other pelvic structures. These include the nerves of the sacral plexus, resulting in foot drop and hamstring compartment weakness (foot drop may also be a result of prolonged squatting in labour, injuring the common peroneal nerve as it traverses the head of the fibula). Bony abnormalities are common, separating or obliterating the symphysis pubis.

Up to half of patients develop upper renal tract abnormalities: scarring of the ureter can cause obstructive uropathies [8]. Up to two thirds of women are rendered amenorrhoeic (their periods stop), either from disorders of the hypothalamic-pituitary axis or from Asherman syndrome (adhesions in the uterus due to scarring). The vagina may be completely destroyed, as may the cervix, causing an obstructive outflow tract resulting in cryptomenorrhoea (women menstruate, but the sloughed blood and tissue don't leave the body).

The continual leakage of urine over the perineal skin can cause local and painful irritation, termed ‘urine dermatitis’. Bladder stones can occur, as women affected by fistula often drink less to try and pass less urine and the concentrated urine can form calculi. The obstetric injury has been termed a ‘field injury’, as the pathology is broad rather than isolated [9]. The resulting range of injuries can be daunting for health professionals who are working with limited resources.

## The Ilorin Experience

A recently published retrospective case note review provides new data on obstetric urogenital fistula in northern Nigeria. Ijaiya and Aboyeji reviewed 34 cases of fistula managed at the University of Ilorin Teaching Hospital over a two-year period [1]. During this period, there were 32,188 deliveries—thus, the incidence of fistula was 1.1 per 1000 live births. The mean age of the women with fistula was 23.9 years, and 32 of the 34 women were illiterate. Half were primiparous. The most common cause of the fistula was obstructed, prolonged labour—the cause in 28 out of the 34 cases. The most common complications of the fistulae were divorce or separation (eight women) and amenorrhoea (seven women).

**What Is Obstetric Fistula?**

‘[Obstetric fistula] usually occurs when a young, poor woman has an obstructed labour and cannot get a Caesarean section when needed. The obstruction can occur because the woman's pelvis is too small, the baby's head is too big, or the baby is badly positioned. The woman can be in labour for five days or more without medical help. The baby usually dies. If the mother survives, she is left with extensive tissue damage to her birth canal that renders her incontinent.’

Source: UNFPA Campaign to End Fistula: “What is Fistula?” (www.unfpa.org/fistula/about.htm).

How does this study compare with other literature on obstetric fistula in Nigeria? First, the incidence reported in the study is lower than that of another hospital study of 22,774 deliveries in Zaria, also in northern Nigeria, which gave an incidence of 3.5 per 1000 deliveries [10]. However, both of these incidence figures are from hospital-based studies, and it is thought that most women do not get to a health facility to deliver their child. So the true incidence of obstetric fistula may well be much higher. In terms of prevalence, it has been estimated that there are up to 800,000 women in Nigeria who have a urogenital fistula from obstructed labour [11]. Second, although the figures given in Ijaiya and Aboyeji's study differ slightly from those of other publications, their study does reconfirm the trends in aetiology and epidemiology of obstetric fistula in the developing world.[Fig pmed-0010002-g002]

**Figure pmed-0010002-g002:**
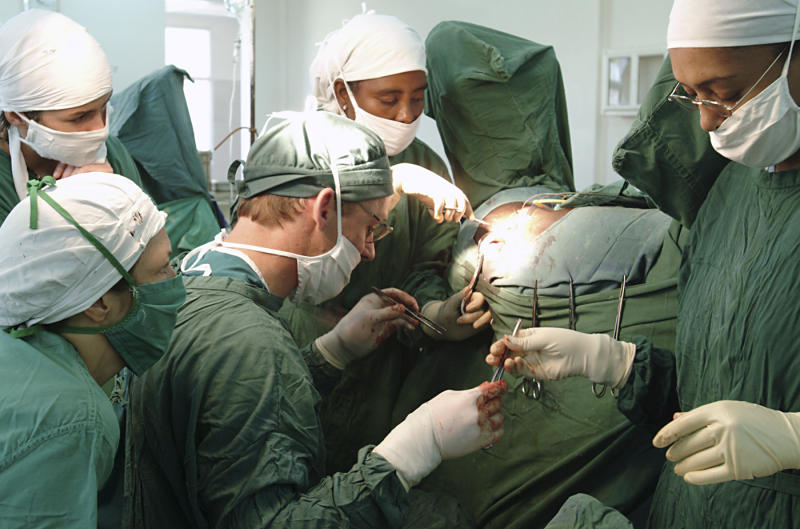
Surgery at the Fistula Hospital, Addis Ababa (Photograph: © 2004 Shaleece Haas. This is an open-access image distributed under the terms of the Creative Commons Attribution License, which permits unrestricted use, distribution, and reproduction in any medium, provided the original work is properly cited.)

## Treatment and Prevention

As in other resource-poor countries, many women with obstetric fistula in Nigeria do not get to a surgeon with expertise in fistula repair. There are, however, a few dedicated professionals in Nigeria helping women with fistula and operating on up to 1,600 women a year [11].

Resource-rich countries were able to eradicate the obstetric fistula almost 100 years ago, but the challenge to resource-poor countries is enormous. There are an estimated 2 million women with fistula in the world, with anywhere between 100,000 and 500,000 new cases developing each year [11]. At the world's current capacity for dealing with the problem, it would take up to 400 years to treat the backlog of patients. Clearly we need many more centres equipped to care for women with fistula. The United Nations Population Fund (UNFPA; www.unfpa.org) and the International Federation of Gynecology and Obstetrics (www.figo.com) are endeavouring to help. UNFPA has already sponsored training workshops on fistula surgery for surgeons and fledgling fistula units in Bangladesh and some parts of Africa.

The obstetric fistula is an entirely preventable condition. Several strategies have been proposed to eradicate this condition in developing countries (Box 1), just as it has been eradicated in the developed world. However, to prevent any new cases of obstetric fistula from occurring, there would need to be 75,000 new emergency obstetric centres built in Africa alone [12]. This would require not only funds, but an appropriate number of trained doctors, nurses, midwives, and support personnel.

**Box 1. The UNFPA's Key Strategies to Address Fistula**

- ‘Postpone marriage and pregnancy for young girls
- ‘Increase access to education and family planning services for women and men
- ‘Provide access to adequate medical care for all pregnant women and emergency obstetric care for all who develop complications
- ‘Repair physical damage through medical intervention and emotional damage through counselling'
  Source: UNFPA Campaign to End Fistula: “Fast Facts” (www.unfpa.org/fistula/facts.htm).

Even if such centres are established, women will need to be convinced of the importance of seeking help without delay for a difficult labour. And then, to be able to receive that help, roads need to be built, transport systems need to be put in place, and communications need to be improved. The obstacles are clearly huge, and with currently very little money and very few professionals available, women with obstetric fistula will sadly be with us for many more years to come.

## Supporting Information

Text S1 Full Text of Ijaiya and Aboyeji's Study [1] (234 KB PDF).

## Abbreviations

UNFPA: United Nations Population Fund

## References

[pmed-0010002-b1] Ijaiya MA, Aboyeji PA (2004). Obstetric urogenital fistula: The Ilorin experience, Nigeria. West Afr J Med.

[pmed-0010002-b2] Hilton P, Ward A (1998). Epidemiological and surgical aspects of urogenital fistula: A review of 25 years experience in south-west Nigeria. Int Urogynecol J Pelvic Floor Dysfunct.

[pmed-0010002-b3] Tomlinson AJ, Thornton JG (1998). A randomized controlled trial of antibiotic prophlyaxis for vesico-vaginal fistula repair. Br J Obstet Gynaecol.

[pmed-0010002-b4] Rangekar NP, Imdad Ail N, Kaul SA, Pathak HR (2000). Role of Martius procedure in the management of urinary-vaginal fistulas. J Amer Col Surg.

[pmed-0010002-b5] Kelly J, Kwast B (1993). Epidemiological study of vesicovaginal fistulas in Ethiopia. Int Urogyn J.

[pmed-0010002-b6] Tahzib F (1983). Epidemiological determinants of vesico-vaginal fistulas. Brit J Obstet Gynecol.

[pmed-0010002-b7] Ampofo K, Out T, Uchebo G (1990). Epidemiology of vesico-vaginal fistulas in northern Nigeria. W Afric J Med.

[pmed-0010002-b8] Lanundoye SB, Bell D, Gill G, Ogunbode O (1976). Urinary tract changes in obstetric vesico-vaginal fistulae: A report of 216 cases studied by intravenous urography. Clin Radiol.

[pmed-0010002-b9] Arrowsmith S, Hamlin EC, Wall LL (1996). Obstructed labor injury complex: Obstetric fistula formation and the multifaceted morbidity of maternal birth trauma in the developing world. Obstet Gynecol Surv.

[pmed-0010002-b10] Harrison KA (1985). Child-bearing, health, and social priorities: A survey of 22,774 consecutive deliveries in Zaria, northern Nigeria. Brit J Obstet Gynecol.

[pmed-0010002-b11] UNFPA (2002). The second meeting of the working group for the prevention and treatment of obstetric fistula, Addis Ababa, 30 October–1 November, 2002. http://www.unfpa.org/upload/lib_pub_file/146_filename_fistula_kgroup02.pdf.

[pmed-0010002-b12] Waaldjik K (1998). Evaluation report XIV on VVF projects in northern Nigeria and Niger.

