# Supplementary material for: Obstetric Fistula in Ilorin, Nigeria
Source: PLoS Med. 2004 Oct 19;1(1):e2. doi: 10.1371/journal.pmed.0010002 (PMC523825; doi:10.1371/journal.pmed.0010002)
Supplement: Text S1 — Full Text of Ijaiya and Aboyeji's Study [1] (234 KB PDF). [file pmed.0010002.sd001.pdf]

Obstetric urogenital fistula: The Ilorin experience, Nigeria

***M. A. Ijaiya and P. A. Aboyeji**
Department of Obstetrics and Gynaecology,
University of Ilorin Teaching Hospital, Maternity Wing,
P. M. B. 1339, Ilorin, Nigeria

Summary

A study of thirty-four cases of obstetric urogenital fistula managed over a ten-year period (1st January, 1989 to 31st December, 1998) at the University of Ilorin Teaching Hospital is reported. The incidence of obstetric urogenital fistula is 1.1 per 1000 births. The condition is associated with illiteracy and poorly supervised delivery. The peak incidence is in 15 - 19 years age group and primipara accounting for 26.5% and 50% respectively.

Prolonged obstructed labour was the most common aetiological factor in 28 (82.4%) of cases. Juxta-cervical region was the most common site of obstetric urogenital fistula. Eight (23.5%) patients were separated/divorced from their husbands.

Obstetric urogenital fistula can be prevented by improving socio-economic condition of the populace, educating the populace to utilize health facilities and advice against teenage pregnancy. Labour should be supervised by trained health personnel and difficult labour referred early to appropriate health care facility.

Keywords: ***Obstetric urogenital fistula, Aetiological factors, Complications, Prevention.***

Résumé

Il s' agit ici d'une étude de trente quatre cas de fistule urogénitale obstétrique faite pendant une période de 10 ans (ler Janvier 1989 au 31 December, 1998) au complexe Hospitalier Universitaire d'Ilorin. L'incidence de la fistule urogénitale obstétrique est de 1, 1 par 1000 couches. Cette condition est liée à l'analphabétisme et à un accouchement mal supervisé. L'incidence la plus graves se retrouve chez le groupe d'âge variant de 15 à 19 ans et chez les primipares, comptant ainsi pour 26.5% et 50% respectivement. Une couche d'enfant prolongée et obstruée est le facteur étiologique commun à 28 (82.4%) des cas. La partie juxta cervicale est le site le plus reconnu à la fistula urogénitale obstétrique. 8 (23, 5%) des patients s' étaient séparés de leurs maris ou avaient divorcé.

La fistule urogénitale obstétrique peut se prévenir par une amélioration des conditions socio-économiques de la population; ainsi il faut éduquer la population à utiliser les facilités sanitaires et déconseiller la grossesseinfantile. La couche devra être supervisée par un personnel sanitaire qualifié. Les couches difficiles devront aussi se référer assez tôt aux agences sanitaires douées.

Introduction

Obstetric urogenital fistula is as old as mankind and has been a constant source of misery to the women affected.[1] It was thought to be an incurable disease until 1675 when the first successful vesico-vaginal fistula repair was performed by Johann Fatio. In 1849, Marion J. Sims, the father of modern gynaeocology, performed his first successful vesico-vaginal fistula (VVF) repair.[2]

Obstetric urogenital fistula is a disease of the developing world.[13] The contributory factors to this high incidence include poverty, illiteracy, ignorance and poor road network with resultant poor utilization of available health facilities hence, it constitutes a major public health problem. It is rarely encountered in industrialised nations because of excellent obstetric care.[4]

It is possible that many of the incidences quoted in texts were under reported because of the shame associated with the condition - as some of the patients may not report in hospital for treatment.

The University of Ilorin Teaching Hospital performs a dual role of a secondary and tertiary health institution. Its catchment areas include Kwara State and some part of Oyo, Osun, Ekiti, Kogi and Niger States. Most of the patients are Yoruba, while Nupe, Ebira, Hausa and Fulani are in the minority.

To the best of our knowledge, there is no published report of urogenital fistula from this Centre. The purpose of this review is to describe our experience with obstetric urogenital fistula in this Centre and to discuss some preventive measures.

Material and Methods

This retrospective study reviewed the thirty-four cases of obstetric urogenital fistula managed at the Department of Obstetric & Gynaeocology of University of Ilorin Teaching Hospital, Ilorin from 1st January 1989 to December 31st, 1998. During the study period, there were 32,188 deliveries.

The case notes of the patients that had obstetric urogenital fistula were retrieved from the Medical Records Department and analysed with respect to age, parity, aetiological factor, anatomical type and complication. The data were analysed by simple percentage.

Results

During the period of study, there were 32,188 deliveries and 34 cases of obstetric urogenital fistula giving an incidence of 1.1 per 1,000 of births.

Thirty-two (94.1%) of the patients were non-literates. The patients were mostly Yoruba 28 (82.4%) while Fulani, Hausa and Nupe were minority with 3 (8.8%), 2 (5.9%) and 1(2.9%) patient(s) respectively. Majority of the patients, 31 (91.1%) attempted or delivered at home.

Table 1 shows age, parity, aetiological factors and anatomical types in patients with obstetric urogenital fistula. The age of the patients ranged between 15 and 43 years with a mean age of 23.9 years. The peak incidence 9(26.5%) was in 15 - 19 years age group. Half, 17 (50.0%) were below 25 years of age. The highest frequency of obstetric urogenital fistula was in primiparous patients, accounting for 17 (50.0%). This

* *Correspondence*

is followed by the grandmultiparous patients 11 (32.4%).

Table 1 Age, parity, aetiological factors and anatomical types in patients with obstetric urogenital fistula

	Variable	No	(%)
i.	Age (Years)		
	15 - 19	9	(26.5)
	20 - 24	8	(23.5)
	25 - 29	6	(17.6)
	30 - 34	7	(20.6)
	35 - 39	3	(8.8)
	40 - 44	1	(2.9)
	**Total**	**34**	**(100)**
**ii**	**Parity**	**No.**	**(%)**
	1	17	(50.0)
	2	1	(2.9)
	3	2	(5.9)
	4	3	(8.8)
	≥5	11	(32.4)
	**Total**	**34**	**(100)**
**iii**	**Aetiological factors**	**No.**	**(%)**
	Prolonged obstructed labour	28	(82.4)
	Ruptured uterus	2	(5.9)
	Caesarean section	2	(5.9)
	Forceps delivery	1	(2.9)
	Caesarean hysterectomy	1	(2.9)
	**Total**	**34**	**(100)**
**iv**	**Anatomical types**	**No.**	**(%)**
	Juxta-cervical fistula	14	(41.2)
	Juxta-urethral fistula	10	(29.4)
	Mid vaginal fistula	5	(14.7)
	Large fistula	4	(11.8)
	Uretero-cervical fistula	1	(2.9)
	**Total**	**34**	**(100)**

Table 2 Complications in patients with obstetric urogenital fistula

Complication	No.	%
Separation/Divorce	8	(23.5)
Amenorrhoea	7	(20.6)
Gynetresia	1	(2.9)

Prolonged obstructed labour was the most common aetiological factor responsible for 28 (82.4%) of cases. The other aetiological factors include ruptured uterus 2 (5.9%), caesarean section 2 (5.9%), forceps delivery 1 (2.9%) and Caesarean hysterectomy 1 (2.9%). Of the 34 cases of obstetric urogenital fistula, 14(41.2%) were juxta-cervical fistula, while 10(29.4%), 5 (14.7%) and 4 (11.8%) were juxta-urethral, midvaginal and large fistula respectively. Uretero-cervical fistula was the least common, 1 (2.9%).

Table 2 shows some of the complications associated with obstetric urogenital fistula. Eight (23.5%) of the patients were divorced (separated) from their husbands, while 7 (20.6%) had amenorrhoea and 1 (2.9%) had gynaetresia from vaginal fibrosis. Some of the patients had multiple complications.

Discussion

The incidence of urogenital fistula in this study is 1.1 per 1,000 births. It is a fair representation of the frequency of the condition in this region since the hospital is the only centre that offers such treatment in this locality. This incidence is within the calculated incidence range of 1 - 2 per 1,000 deliveries in Northern Nigeria.[1]

Women aged between 15 and 19 years and primipara have the highest frequency of obstetric urogenital fistula in this study. The finding is similar to other reports.[2-7] The reason is that the pelvis is not fully developed in teenagers to allow for normal vaginal delivery and this could explain the high frequency of prolonged obstructed labour as the leading aetiological factor to the development of obstetric urogenital fistula. This is also the finding of other authors.[3,4,7,8]

In this study, juxtacervical fistula was the most common type of obstetric urogenital fistula encountered. This is in contrast to previous report[4] on the subject where it was found that juxta-urethral was the most common fistula following prolonged obstructed labour.

It is not surprising that psycho-social complication (separation/divorce) was the leading complication of this condition in this study. The magnitude of this complication has also been emphasised by other authors.[2,4,6,8] The patients are deserted by their husbands because of the smell of urine, which makes them socially unacceptable.

As it is stated earlier on, prolonged obstructed labour remains the major cause of obstetric urogenital fistula in this environment. Preventive measures include improving socio-economic condition of the community to prevent pelvic bone abnormalities from childhood malnutrition. Emphasis should also be laid on female education. This delays age at marriage, allows for better utilization of available health facilities for antenatal care, hospital confinement and family planning. Early marriage should also be discouraged and good road network should be provided for easy transportation of patients.

The district and secondary hospitals should be strengthened to enable caesarean section to be done. Where it is not possible, high risk patients should be identified and referred to appropriate Centre. Trained health personnel should supervise labour and partograph should be used at all levels of health care delivery with prompt referral when necessary. Obstetric manipulation/operation should be performed by those skilled in the art.

Conclusion

Obstetric urogenital fistula is a preventable condition but still constitutes a major public health problem in developing countries. The women afflicted with this condition eventually become social outcast and demoralised. Individual, governmental and non-governmental efforts are needed to prevent the occurrence of this condition and rehabilitation of the affected patients.

Acknowledgement

We wish to thank all the consultant staff for allowing us to review their patients' case notes.

References

1. Waaldijk K and Armiya'u YD: The obstetric fistula: A major public health problem still unsolved. Int. Urogynecol J. 1993; 4: 126 - 128.

2. Thomas Margolis and Lane Mercer: Vesico-vaginal fistula. Obstet. Gynecol. Surv. 1994; 49: 840 - 844.

3. Ekwempu CC: Fistula. In textbook of Obstetrics and Gynaecology for medical students Vol 1; Akin Agboola (Ed.) University Series Educational Pub. Ltd. 1988; pp. 46 - 59.

4. Lawson JB: Injuries of the urinary tract. In obstetrics and gynaecology in the tropics and developing countries. JB Lawson, DB Stewart (Ed.) and Edward Arnold, London pub. Ltd. 1967; pp 481-522.

5. Ekele BA and Dikko A A: Urogenital fistulae in Sokoto. Trop. J. Obstet. Gynaecol. 1997; 14: 43 - 45.

6. Kofi Ampofo, Theresa Otu and Gilbert Ilelebo: Epidemiology of Vesico-vaginal fistula in Northern Nigeria. West Afr. J. Med.; 1990; 9: 98 - 102.

7. Javaid H and Rizvi: Genital fistulate - A continuing Tragedy, J. Obstet. Gynaecol. Res.; 1999; Vol. 25: 1- 7.

8. Chassar Moir J: J. Marion Sims and the VVF Then and Now. In vesico-vaginal fistula: J Chassar Moir (Ed.) Second Edition, Morrison & Gibb pub. ltd. 1967; pp. 1 - 16.
